# Multi-state study of *Enterobacteriaceae* harboring extended-spectrum beta-lactamase and carbapenemase genes in U.S. drinking water

**DOI:** 10.1038/s41598-019-40420-0

**Published:** 2019-03-08

**Authors:** Windy D. Tanner, James A. VanDerslice, Ramesh K. Goel, Molly K. Leecaster, Mark A. Fisher, Jeremy Olstadt, Catherine M. Gurley, Anderson G. Morris, Kathryn A. Seely, Leslie Chapman, Michelle Korando, Kalifa-Amira Shabazz, Andrea Stadsholt, Janice VanDeVelde, Ellen Braun-Howland, Christine Minihane, Pamela J. Higgins, Michelle Deras, Othman Jaber, Dee Jette, Adi V. Gundlapalli

**Affiliations:** 1 University of Utah, Salt Lake City, UT USA; 2 VA Salt Lake City Healthcare System, Salt Lake City, UT USA; 3 Wisconsin State Laboratory of Hygiene, Madison, WI USA; 4 Arkansas Department of Health Public Health Laboratory, Little Rock AR, USA; 5 Illinois Department of Public Health, Carbondale, IL USA; 6 Illinois Department of Public Health, Chicago, IL USA; 7 Illinois Department of Public Health, Springfield, IL USA; 8 Wadsworth Center, Albany, NY USA; 9 Pennsylvania Department of Environmental Protection, Harrisburg, PA USA; 10 Weber Basin Water Conservancy District, Layton, UT USA; 11 Utah Public Health Laboratory, Taylorsville, UT USA; 12 Davis County Health Department, Clearfield, UT USA

## Abstract

Community-associated acquisition of extended-spectrum beta-lactamase- (ESBL) and carbapenemase-producing *Enterobacteriaceae* has significantly increased in recent years, necessitating greater inquiry into potential exposure routes, including food and water sources. In high-income countries, drinking water is often neglected as a possible source of community exposure to antibiotic-resistant organisms. We screened coliform-positive tap water samples (n = 483) from public and private water systems in six states of the United States for *bla*_CTX-M_, *bla*_SHV_, *bla*_TEM_, *bla*_KPC_, *bla*_NDM_, and *bla*_OXA-48_-type genes by multiplex PCR. Positive samples were subcultured to isolate organisms harboring ESBL or carbapenemase genes. Thirty-one samples (6.4%) were positive for *bla*_CTX-M_, ESBL-type *bla*_SHV_ or *bla*_TEM_, or *bla*_OXA-48_-type carbapenemase genes, including at least one positive sample from each state. ESBL and *bla*_OXA-48_-type *Enterobacteriaceae* isolates included *E. coli, Kluyvera*, *Providencia*, *Klebsiella*, and *Citrobacter* species. The *bla*_OXA-48_-type genes were also found in non-fermenting Gram-negative species, including *Shewanella, Pseudomonas* and *Acinetobacter*. Multiple isolates were phenotypically non-susceptible to third-generation cephalosporin or carbapenem antibiotics. These findings suggest that tap water in high income countries could serve as an important source of community exposure to ESBL and carbapenemase genes, and that these genes may be disseminated by non-*Enterobacteriaceae* that are not detected as part of standard microbiological water quality testing.

## Introduction

Antibiotic-resistant infections are responsible for an estimated 2 million illnesses and 23,000 deaths in the United States each year^1^. The rising prevalence of multidrug-resistant bacteria is especially alarming, as infections with these organisms have led to increasing use of broad spectrum antibiotics such as third and fourth generation cephalosporin and carbapenem antibiotics^1,2^. Enzymes such as extended-spectrum beta-lactamases (ESBLs) and carbapenemases can render these antibiotics ineffective. *Enterobacteriaceae* harboring these enzymes are ranked among the most urgent antibiotic resistance threats according to the U.S. Centers for Disease Control and Prevention and the World Health Organization^1,3^. Additionally, genes encoding these enzymes are often found on mobile genetic elements that can be transferred horizontally to other bacterial species^4^.

ESBL- and carbapenemase-producing bacteria are commonly associated with healthcare contact^1^; however, community-associated infections have significantly increased in recent years^5^. Between 2009 and 2011, the occurrence of ESBL-producing bacteria in community-associated infections increased from 3.1% to 12.6%, while the occurrence in hospital-associated infections remained the same^5^. It is estimated that as many as two-thirds of all ESBL-producing *Enterobacteriaceae* infections are community-associated^5,6^. In 2013, the U.S. Centers for Disease Control warned that spread of carbapenem-resistant *Enterobacteriaceae* (CRE) into the community could reasonably be expected, as experienced with ESBLs^1^. A 2017 review on CRE in the community noted that the prevalence of CRE among U.S. community-associated study samples ranged from 5.6 to 10.8%^7^.

ESBL and carbapenemase genes are frequently found in *Enterobacteriaceae* such as *Klebsiella pneumoniae*, *Escherichia coli*, *Enterobacter cloacae*, and *Citrobacter* species^4^, but can also be found in non-fermenting Gram-negative species^8^. These bacteria are most commonly spread via fecal-oral transmission routes, including direct transmission (e.g. via hands) and indirect transmission (e.g. via the environment). *Enterobacteriaceae*, including ESBL- and carbapenemase-producing strains, have been reported in a number of environmental compartments including food, animals, surface waters, and drinking water^9–11^. Studies reporting carbapenemase or ESBL genes in drinking water have largely been performed in low-income countries^9,12^. In high income countries, reports of ESBL- and carbapenemase-producing bacteria in drinking water have been limited to single-cases or intrinsic genes in nonpathogenic environmental bacterial species^10,13^, but comprehensive studies are lacking.

In clinical infections, ESBL and carbapenemase genes are most frequently found in *Enterobacteriaceae* species, but these organisms are typically uncommon in chlorinated public drinking water supplies in high income countries. To potentially increase detection of ESBL and carbapenemase genes in U.S. drinking water, we targeted water samples testing positive for *Enterobacteriaceae* (coliform bacteria). The objectives of our study were (1) to determine whether ESBL- and carbapenemase-producing genes are present in U.S. drinking water samples that have tested positive for *E. coli* or total coliform bacteria, (2) to describe the sample and water system characteristics associated with samples testing positive for ESBL or carbapenemase genes, and (3) to determine if the ESBL and carbapenemase genes are present in viable bacteria isolated from the coliform-positive water samples.

## Methods

### Water sample collection and coliform testing

Between July 2015 and November 2016, regulatory and investigational drinking water samples testing positive for *E. coli* or total coliform bacteria (*Enterobacteriaceae*) were acquired from multiple local and state public health laboratories that perform regulatory water quality testing for water utilities in their state or county. Participating laboratories included the state public health and environmental laboratories in Wisconsin (99 samples), New York (22 samples), Pennsylvania (69 samples), Illinois (125 total samples from three laboratories), Arkansas (64 samples), and Utah. Additionally, one water utility and one county health laboratory in Utah supplied coliform-positive samples (total of 104 Utah samples). Sample acquisition was opportunistic, and may not have consistently included all coliform-positive samples from each site. Private well samples were only included in the study if positive for *E. coli*, to avoid testing a high proportion of private well samples, which frequently test positive for coliform bacteria. Drinking water samples collected for regulatory water quality testing are required to be 100 milliliters in volume and must be preserved with sodium thiosulfate and tested at the laboratory within 30 hours of collection, preferably held at a temperature between 0 and 10 degrees Celsius during transport^14^.

**Figure 1:**
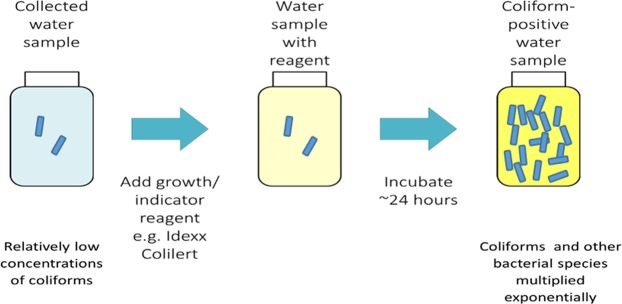
Water sample coliform screening process.

Upon arrival to each public health laboratory location, drinking water samples were tested using a conventional enzyme-substrate method that indicates the presence of *E. coli* and total coliform bacteria (Fig. 1)^15^. The substrate reagent includes a nutrient medium that creates culture conditions in the water sample and promotes bacterial growth and has chromogenic and fluorogenic indicators to detect total coliforms and *E. coli*, respectively. Samples were tested in either a presence/absence format using the original 100-mL collection vessel, or in a quantitative format by dispensing the 100 mL sample into a multi-well tray (Quanti-Tray, IDEXX, Westbrook, ME) for quantification by the most-probable-number technique. When *E. coli* and/or coliform bacteria were detected in cultured presence/absence samples, 1 mL of the positive enriched sample was placed in a sterile cryovial with 1 mL 40% glycerol (final concentration 20% glycerol). If a sealed Quanti-Tray sample was positive, the back of the tray was disinfected and a sterile syringe was used to extract the culture liquid from positive wells. When multiple wells indicated coliforms, a composite 1 mL of enriched sample was produced by extracting liquid from several wells, and the composite was mixed with glycerol as described above. All samples were cryogenically frozen, and samples from locations other than Utah were shipped on dry ice overnight to the research laboratory in Salt Lake City, Utah.

### Resistance gene detection and PCR amplicon sequencing

Upon arrival to the research laboratory, preserved samples were screened for the three ESBL genes most common in the U.S. (*bla*_SHV_, *bla*_TEM_, and *bla*_CTX_) by multiplex PCR, as previously described^16^. Samples were also screened for the three most common carbapenemase genes (*bla*_OXA-48_-type, *bla*_NDM_, and *bla*_KPC_) by a separate multiplex PCR^17^. Primer sequences are available in Supplemental Table 1. Four microliters of the preserved sample culture was directly used as a template for the PCR in a total reaction volume of 25 µL. Positive controls included American Type Culture Collection isolate BAA-2146 for *bla*_SHV_, *bla*_TEM_, *bla*_CTX-M_, and *bla*_NDM_ detection, a clinical KPC-producing *K. pneumoniae* for *bla*_KPC_ detection, and an OXA-48-producing *Shewanella* for *bla*_OXA-48_ detection. PCR products were run on a 1% agarose gel, and amplicon from samples putatively positive for any of the genes of interest were sequenced by the Sanger method using the forward and reverse primers used for PCR screening. If samples were positive for multiple genes, PCR was repeated with individual primer pairs in separate reactions, followed by amplicon sequencing. Sequences were searched against the Comprehensive Antibiotic Resistance Database (CARD) and the GenBank (BLASTn) database to confirm the presence of one of the six target ESBL or carbapenemase genes. SHV and TEM variants identified by these databases were compared against the Lahey Clinic designation of ESBL-type *bla*_SHV_ and *bla*_TEM_ genes^18^. Lack of specificity of the multiplex CTX-M primer can result in amplification of several other beta-lactamase genes, such as *bla*_OXY_^16^; as a result, chromatograms from all CTX-M –positive amplicon sequences were closely inspected to see if multiple genes may have been present in the sample. In the case of mixed chromatograms, samples were tested as described above using a CTX-M group-specific multiplex PCR^19^ (Supplemental Table 1). Samples confirmed as positive for any of the target genes by Sanger sequencing were subsequently tested for the specific gene using primers that amplified larger segments of the genes to better identify the specific alleles^20–23^.

**Table 1:** Characteristics of sources of samples positive for *bla*_CTX-M_, *bla*_OXA-48_-type, and putative *bla*_SHV_ or *bla*_TEM_ genes; ^a^Gene variant or cluster with greatest homology in GenBank; ^b^CPWS: Community public water system, NCPWS: Non-community public water system; ^c^Samples collected from the same water systems: A1 and A2; D1, D2, and D3; E2, E5, and E6; ^d^100% nucleotide homology to uncultured bacterium clones; ^e^Gene sequence is similarly homologous with both ESBL and non-ESBL *bla*_TEM_ genes, but exhibits an ESBL phenotype ^f^Blended water is a composite of ground water and treated surface water.

Sample ID	Gene variant or group with highest DNA sequence similarity^a^	Gene homology with reference sequence	Protein homology with reference sequence	GenBank Nucleotide Reference (GenBank Protein reference)	Organism	*E. coli* present/absent in sample	System type^b^	Water source	GenBank Accession Number
A1^c^	CTX-M-205	79%	86%	MG028655 (ATJ25945.1)	*Citrobacter freundii*- complex	Absent	CPWS	Ground	MG560059
A2^c^	CTX-M-205	80%	88%	MG028655	*Citrobacter freundii*- complex	Present	CPWS	Ground	MG560060
A3a	CTX-M-40 or CTX-M-63	84%	94%	NG_048991 NG_049014	*Kluyvera georgiana*	Present	Private	Ground	MG560061
A3b	OXA-181	100%	100%	CP023897	*Acinetobacter baumannii complex*	Present	Private	Ground	MG560077
A4	CTX-M-40 or CTX-M-63	98%	99%	NG_048991 NG_049014	*Kluyvera georgiana*	Absent	CPWS	Ground	MG560062
A5	CTX-M-40 or CTX-M-63	84%	94%	NG_048991 NG_049014	*Kluyvera georgiana*	Absent	CPWS	Ground	MG560063
A6	CTX-M-9 family	86%^d^	95%	NG_049028.1	Not Isolated	Absent	CPWS	Surface	MG560055
B1	CTX-M-10 or CTX-M-34	100%	100%	NG_048898 NG_048984	*Kluyvera* species	Present	Private	Ground	MG560064
B2	CTX-M-40 or CTX-M-63	96%	95%	NG_048991 NG_049014	Not Isolated	Present	Private	Unknown	MG560056
B3a	CTX-M-12	100%	100%	DQ821704	Not Isolated	Present	Private	Ground	MG560057
B3b	OXA-48b	100%	100%	KU820807	Not Isolated	Present	Private	Ground	MG560068
B4	OXA-48b	100%	100%	KU820807	*E. coli*	Present	Private	Ground	MG560078
B5	OXA-181	85%^d^	93%	KJ620504	*E. coli*	Present	Private	Ground	MG560079
B6	OXA-48b	100%	100%	KU820804	*Shewanella* species	Present	Private	Ground	MG560080
B7	CTX-M-3	100%	100%	KU200455	*Klebsiella oxytoca*	Present	Private	Ground	MG560065
B8	OXA-181	85%	94%	KJ620504	Not Isolated	Present	Private	Ground	MG56006
B9	OXA-48b	100%	100%	KU820804	*Shewanella* species	Present	Private	Ground	MG560081
B10	OXA-252	100%	100%	CP022089	Not Isolated	Present	Private	Ground	MG560070
B11a	OXA-48b	99%	99%	KC902850	*Shewanella putrefaciens;*	Absent	NCPWS	Ground	MG560082
B11b	OXA-48b	99%	99%	KC902850	*Pandoraea sputorum*	Absent	NCPWS	Ground	MG560083
B12	TEM-1 variant^e^	99%	99%	KU664682.1	*E. coli*	Present	Private	Ground	MG560088
B13	OXA-48b or OXA-252	100%	100%	KU820804 NG_050608.1	*Shewanella* species	Present	Private	Ground	MG560084
C1	CTX-M-3	99%	99%	Y10278	Not Isolated	Absent	CPWS	Surface	MG560058
C2	CTX-M-2-like	89%	96%	KX377894	*Kluyvera ascorbata*	Absent	CPWS	Ground	MG560066
D1^c^	OXA-48b	100%	100%	KU820807	*Providencia rettgeri*	Absent	NCPWS	Ground	MG560085
D2^c^	OXA-252	100%	100%	NG_050608	Not Isolated	Present	NCPWS	Ground	MG560071
D3^c^	OXA-199	99%	99%	NG_049495	Not Isolated	Present	NCPWS	Ground	MG560072
E1a	CTX-M-36	99%	100%	NG_048986	*Klebsiella oxytoca*	Absent	CPWS	Blend^f^	MG560067
E1b	OXA-48b	100%	100%	KU820807	Not Isolated	Absent	CPWS	Blend^f^	MG560073
E2^c^	OXA-48b	100%	100%	KU820807	Not Isolated	Absent	CPWS	Blend^f^	MG560074
E3	OXA-252	100%	100%	NG_050608	*Pseudomonas* species	Absent	CPWS	Blend^f^	MG560075
E4	OXA-48b	100%	100%	KU820802	Not Isolated	Absent	CPWS	Blend^f^	MG560076
E5^c^	OXA-48b	100%	100%	KU820807	*Pseudomonas koreensis*	Absent	CPWS	Blend^f^	MG560086
E6^c^	OXA-252	100%	100%	KU820800	*Pseudomonas putida*	Absent	CPWS	Blend^f^	MG560087
F1	SHV-38	100%	100%	NG_050077	*Klebsiella pneumoniae*	Present	NCPWS	Ground	MG560089

### Bacterial isolation and identification

Bacteria carrying the target resistance genes were isolated through selective and non-selective culture of the preserved samples as previously described^24,25^. Samples confirmed for the presence of *bla*_TEM_, *bla*_SHV_, or *bla*_CTX-M_ genes were plated on CHROMagar Orientation^TM^ agar plates (DRG International, Springfield, NJ) with and without a proprietary ESBL supplement. Approximately 50 µL of the preserved sample was spread onto each plate, and cefotaxime (30 µg), ceftazidime (30 µg), and aztreonam (30 µg) disks were placed on the non-selective CHROMagar plates. After overnight incubation, colonies on the ESBL CHROMagar plate and colonies falling within a distinct zone forming around the disks on the non-selective plate were tested by PCR for *bla*_TEM_, *bla*_SHV_, or *bla*_CTX-M_ genes using the primer set from the ESBL multiplex PCR.

For samples confirmed as having a *bla*_OXA-48_-type gene, 50 µL of preserved sample was spread onto an mSuperCARBA^TM^ plate (DRG International, Springfield, NJ) and a non-selective CHROMagar Orientation^TM^ plate (DRG International, Springfield, NJ) with a temocillin disk (Rosco, Taastrup, Denmark). In cases where the *bla*_OXA-48_-type producer could not be isolated by direct plating, 20 µL of the original culture sample was added to a Luria broth with 0.5, 1, 2, and 4 µg/mL imipenem. Broths testing positive by PCR were plated as described above and to sheep’s blood agar with 0.125 µg/mL ertapenem. Colonies growing on the mSuperCARBA^TM^ plate and colonies growing within the distinct zone that formed around the temocillin disk were tested for *bla*_OXA-48_-type genes using the OXA-48 primer set from the carbapenemase gene multiplex PCR and confirmed by Sanger sequencing of the amplicon. Bacterial species identification was performed by MALDI-TOF (Biotyper, Bruker, Bellerica, MA) using the full spectral library which is composed of spectra representing roughly 2750 species of microorganisms from approximately 470 genera^26^.

### Susceptibility testing

Minimum inhibitory concentrations of isolates were determined using Thermo Scientific^TM^ Sensititre^TM^ Extended Spectrum Beta-lactamase plates (Trek Diagnostic Systems, Inc., Independence, OH) according to manufacturer’s directions. For isolates in which the genes were lost upon subculture, fresh colonies from the original sample were used in preparation of the antimicrobial susceptibility testing inoculum, if sufficient growth was present.

### Supplementary sample data

Laboratories supplying coliform-positive samples also provided limited data on sample characteristics such as water source (e.g. groundwater, [treated] surface water, or blended), sample type (regulatory or investigational), and system type (e.g. community public water system [CPWS], non-community public water system [NCPWS], or private well, as defined by the U.S. Environmental Protection Agency)^27^. Sample results were described with respect to these characteristics. Statistical analyses to test specific hypotheses or make statements of inference would have been inappropriate because the sample collection was opportunistic.

## Results

### Sample characteristics

A total of 483 non-duplicate samples, representing 361 public and private water systems, were collected. Of the 483 samples, 27% were from private water systems, 31% were from CPWSs, and 42% were from NCPWSs. Spring water sources comprised 5% of the samples, while surface and ground water sources made up 9% and 78%, respectively. Approximately 6% of the samples were blended, meaning that they were from systems where both groundwater and treated surface water were supplied to consumers during periods of high demand.

### PCR detection of ESBL and carbapenemase genes and amplicon sequencing

The 483 samples were screened for *bla*_TEM_, *bla*_SHV_, *bla*_CTX-M_, *bla*_KPC_, *bla*_NDM_, and *bla*_OXA-48_-type genes. Sixty-four samples appeared to be positive for *bla*_CTX-M_ by initial PCR and agarose gel screening; however, comparison of PCR amplicon sequences to the GenBank database revealed that the majority of the putative *bla*_CTX-M_ positives were attributed to extended-spectrum beta-lactamase genes that are typically chromosomally-encoded in various *Enterobacteriaceae* species, including *bla*_RAHN_ (*Rahnella aquatilis*), *bla*_FONA_ (*Serratia fonticola*), *bla*_OXY_ (*Klebsiella oxytoca*), *bla*_SMO_ (strain RUS)^28^, and *Citrobacter amalonaticus* class-A beta lactamase. Because these were not target genes in the study, we did not pursue any further analysis of these samples.

Thirty-one samples (6.4%) from twenty-six (7.2%) of the water systems were positive for the target *bla*_CTX-M_, *bla*_OXA-48_-type, or ESBL-type *bla*_TEM_ or *bla*_SHV_ genes. Thirteen of the samples (2.7%) from twelve different water systems (3.3%) were confirmed to have *bla*_CTX-M_ genes based on amplicon sequences. Thirteen (2.7%) and ten (2.1%) samples were positive for *bla*_SHV_ and *bla*_TEM_ genes, respectively. One SHV-positive amplicon sequence most closely matched an ESBL-type *bla*_SHV_ (*bla*_SHV-38_) and one TEM-positive amplicon sequence closely matched both ESBL- and non-ESBL-type *bla*_TEM_ genes. The carbapenemase multiplex PCR screen did not reveal any NDM- or KPC-positive samples; however, *bla*_OXA-48_-type genes were detected in 19 samples (3.9%) from 15 (4.2%) of the water systems. The samples with *bla*_OXA-48_-type genes were from four states and represented between 2–7% of samples from those states. Three of the samples that tested positive for *bla*_OXA-48_-type genes were also positive for *bla*_CTX-M_ genes and were each from different states. At least one ESBL or carbapenemase gene was detected in all six states comprising between 1% and 15% of the coliform-positive samples tested from each state. Of the 185 water systems with repeat samples, six had samples that tested positive for at least one ESBL or carbapenemase gene; half of these water systems had only one positive sample and one water system had all three samples positive.

### Isolation of ESBL- or carbapenemase-producing bacteria and antimicrobial susceptibility testing

Bacteria carrying target ESBL or carbapenemase genes were isolated from 21 of the 31 positive samples (Table 1). The *bla*_CTX-M_ gene was detected in *Klebsiella oxytoca, Citrobacter freundii* complex, *Kluyvera ascorbata* and *Kluyvera georgiana*, the latter two being the progenitor of *bla*_CTX-M_ genes. The ESBL-type *bla*_SHV-38_ gene was present in *Klebsiella pneumoniae* and a *bla*_TEM_ variant gene exhibiting an ESBL phenotype was present in *E. coli*. Species carrying *bla*_OXA-48-_type genes included *E. coli*, *Providencia rettgeri*, *Acinetobacter baumannii* complex, *Pseudomonas putida*, *Pseudomonas koreensis, Pandoraea sputorum*, *Shewanella putrefaciens*, and other *Shewanella* (n = 3) and *Pseudomonas* (n = 1) that could not be identified at the species level. The stability of ESBL and *bla*_OXA-48-_type genes were noted based on the number of subcultures where the resistance gene could still be detected in culture. The ESBL genes were very stable and were still detected after two or more subcultures. The *bla*_OXA-48-_type genes were typically lost after one subculture in non-*Shewanella* non-fermenting Gram-negative rods, and after two or more subcultures in *Enterobacteriaceae*, with or without selective pressure from imipenem concentrations ranging from 0.125 ug/mL to 4 ug/mL. Nucleic acid sequences of the PCR amplicon from isolates and from positive samples where an isolate could not be recovered are available in GenBank (Table 1). Antimicrobial susceptibility testing results for selected isolates are presented in Table 2.

**Table 2:** Minimum inhibitory concentrations (MIC) (µg/mL) of isolates to selected antibiotics by broth microdilution.

Sample ID	Organism (gene type)	ESBL pheno-type^a^	TAZ	FOT	FAZ	FEP	FOX	CEP	POD	AXO	IMI	MEM	GEN	AMP	CIP	P/T4
A1	*C. freundii*-complex (CTX)	No	≤0.25	≤0.25	16*	≤1	8*	>16*	1	≤1	1	≤1	≤4	≤8*	≤1	32/4
A2	*C. freundii*-complex (CTX)	No	≤0.25	0.5	>16*	≤1	8*	>16*	1	≤1	≤0.5	≤1	≤4	16*	≤1	≤4/4
A3a	*K. georgiana*^b^ (CTX)	No	≤0.25	≤0.25	>16	≤1	≤4	>16	2	≤1	≤0.5	≤1	≤4	>16	≤1	≤4/4
A3b	*A. baumannii* complex (OXA-48)	Yes	8	8	>16	2	64	>16	8	8	≤0.5	≤1	≤4	16*	≤1	64/4
A4	*K. georgiana*^b^ (CTX)	No	≤0.25	≤0.25	>16	≤1	≤4	>16	1	≤1	4	≤1	≤4	>16	≤1	64/4
A5	*K. georgiana*^b^ (CTX)	No	0.5	0.5	>16	≤1	≤4	>16	2	2	≤0.5	≤1	≤4	>16	≤1	≤4/4
B1	*Kluyvera* species^b^ (CTX)	Yes	32	2	>16	8	8	>16	16	4	≤0.5	≤1	≤4	>16	≤1	≤4/4
B4	*E. coli* (OXA-48)	Yes	8	8	16	2	64	>16	0.5	≤1	≤0.5	≤1	8	≤8	≤1	>64/4
B5	*E. coli* (OXA-48)	Yes	8	4	≤8	>16	≤4	>16	1	≤1	1	≤1	≤4	≤8	≤1	>64/4
B6	*Shewanella* species^b^ (OXA-48)	No	≤0.25	2	>16	≤1	≤4	>16	≤0.25	≤1	≤0.5	>8	≤4	16	≤1	≤4/4
B7	*K. oxytoca* (CTX)	No	1	≤0.25	≤8	≤1	≤4	≤8	≤0.25	≤1	≤0.5	≤1	≤4	>16*	≤1	≤4/4
B11	*S. putrefaciens*^b^ (OXA-48)	Yes	0.5	2	>16	≤1	8	≤8	≤0.25	64	≤0.5	4	≤4	16	≤1	≤4/4
B12	*E. coli* (TEM)	Yes	8	8	≤8	>16	8	>16	16	4	≤0.5	≤1	≤4	>16	≤1	>64/4
B13	*Shewanella* species^b^ (OXA-48)	No	≤0.25	≤0.25	>16	≤1	≤4	>16	≤1	≤1	≤0.5	2	16	≤8	≤1	≤4/4
C2	*K. ascorbata*^b^ (CTX)	No	≤0.25	≤0.25	>16	≤1	≤4	>16	1	≤1	≤0.5	≤1	≤4	≤8	≤1	≤4/4
D1	*P. rettgeri* (OXA-48)	No	0.5	≤0.25	>16*	2	64	>16*	≤0.25	≤1	≤0.5	≤1	≤4	≤8*	≤1	≤4/4
E1a	*K. oxytoca* (CTX)	No	0.5	1	≤8	≤1	≤4	>16	4	16	≤0.5	≤1	≤4	≤8*	≤1	≤4/4
E3	*Pseudomonas* species^b^ (OXA)	No	1	2	>16	≤1	>64	>16	2	8	≤0.5	≤1	≤4	>16	≤1	≤4/4
F1	*K. pneumoniae* (SHV)	Yes	64	4	≤8	≤1	≤4	>16	4	≤1	≤0.5	≤1	16	>16*	≤1	>64/4

TAZ: ceftazidime, FOT: cefotaxime, FAZ: cefazolin, FEP: cefepime, FOX: cefoxitin, CEP: cephalothin, POD: cefpodoxime, AXO: ceftriaxone, IMI: imipenem, MEM: meropenem, GEN: gentamicin, AMP: ampicillin, CIP: ciprofloxacin, P/T4: piperacillin/tazobactam. Cells marked with a * indicate antibiotics to which the organism is considered intrinsically resistant according to the 2019 Clinical and Laboratory Standards Institute (CLSI) M100 ED29:2019 document^34^; ^a^An ESBL phenotype is defined as an bacterial phenotype that exhibits a ≥3 two-fold concentration decrease in a MIC for ceftazidime or cefotaxime tested in combination with clavulanate vs the MIC of ceftazidime or cefotaxime when tested alone. Although the result is listed for all organisms in Table 2, this test is intended for *Klebsiella pneumoniae*, *Klebsiella oxytoca*, *E. coli*, and *Proteus mirabilis* per CLSI m100^34^; ^b^Indicates organisms for which an intrinsic resistance profile is not available in the CLSI m100 document.

### Resistance gene presence and tap water source characteristics

The *bla*_CTX-M_, *bla*_OXA-48_-type, or ESBL-type *bla*_TEM_ or *bla*_SHV_ genes were found in 5.6% of samples from ground water sources and 7.0% of samples from treated surface water sources, and from 10.0% of private and 5.1% of public water systems: 8.7% of CPWS and 2.5% of NCPWS samples. Overall, 6.8% of the coliform-positive samples from public water systems also tested positive for *E. coli*; however, when considering those samples that tested positive for an ESBL or *bla*_OXA-48_-type gene, 22.2% were positive for *E. coli*.

## Discussion

Community-associated antibiotic-resistant infections caused by ESBL- and carbapenemase-producing bacteria have increased significantly in the U.S. over the past decade. Many possible transmission routes have been studied; but in high-income countries, drinking water has not been adequately assessed as a potential source. We detected ESBL genes (*bla*_CTX-M_, *bla*_TEM_, or *bla*_SHV_) or *bla*_OXA-48_-type carbapenemase genes in more than 6% of the coliform-positive U.S. drinking water samples screened. Coliform-positive drinking water samples were targeted for testing because ESBL and carbapenemase genes are most commonly found in *Enterobacteriaceae* in clinical settings.

Non-*E. coli* coliforms, such as *Klebsiella*, *Citrobacter*, *Enterobacter*, and *Serratia* species are considered to be non-pathogenic by regulatory agencies and are primarily used as indicators of potential fecal contamination or water distribution system breaches. However, it is important to consider that these species are also some of the most common carriers of ESBL and carbapenemase genes, regardless of pathogenicity^24^. In 2015, the presence of coliform bacteria was reported in 1909 U.S. water systems serving over 10 million in total people, although audits and other compliance reports have found that underreporting of drinking water contaminants in public water systems is likely a widespread issue^29^. Coliform bacteria are even more common in private wells, which supply approximately 15% of the U.S. population with drinking water^30^.

This study utilized a convenience sample of coliform-positive water samples from state and local public health and environmental laboratories to increase the potential for finding ESBL- or carbapenemase-producing *Enterobacteriaceae*, not to estimate the overall frequency of occurrence of these genes in U.S. water supplies. The study design limits the conclusions that can be drawn regarding the overall prevalence of these genes in U.S. water systems and limits access to some information associated with individual samples, such as the type of water treatment used by the water systems. There is also a slight possibility that some coliform-positive samples resulted from accidental contamination during original sample collection; however, regulatory samples are typically collected by public water system personnel trained in proper sample collection technique.

The true prevalence of the target ESBL and carbapenemase genes in these samples may be underestimated due to testing limitations. A very small proportion of the preserved water sample culture was tested, potentially missing ESBL and carbapenemase genes present in low copy numbers in the samples. Additionally, DNA was not extracted from the preserved samples prior to PCR testing, and PCR inhibitors may have been present, affecting gene detection. We were also only able to determine presence or absence of the target resistance genes and could not assess the abundance in the original water sample due to the culture step in the coliform screening process.

ESBL and carbapenemase genes other than the target genes were also clearly present in these water samples. Some of these genes may have been carried by the bacterial isolates in addition to the target ESBL or carbapenemase genes that were detected, potentially contributing to any observed phenotype. The majority of these non-target genes are known to be chromosomally-located and carried by nonpathogenic bacterial species; however, some, such as *bla*_OXY_, can be plasmid-mediated, and may still be a cause for clinical concern^31^. The *bla*_CTX-M_ and *bla*_OXA-48_-type genes also arise from relatively nonpathogenic bacteria that are commonly found in water sources (*Kluyvera* and *Shewanella* species, respectively), but have been widely disseminated to other bacterial species via mobile genetic elements. In this study, *bla*_OXA-48_-type genes in non-*Shewanella* species were lost in subsequent subcultures, a phenomenon also observed with non- fermenting Gram-negative rods carrying the *bla*_NDM-1_ carbapenemase gene isolated from New Delhi drinking water^9^.

Bacterial isolates were identified using the full spectral Bruker MALDI-TOF library used by diverse laboratories. Despite the broad organism coverage, it is possible that some environmental species may have limited representation in this system. In our study, all of the *bla*_CTX-M_ carriers were identified as *Enterobacteriaceae*, with approximately half being classified as *Kluyvera* species. *Enterobacteriaceae* carrying *bla*_OXA-48-_type genes were also isolated from some water samples. The *bla*_OXA-48_-like genes were also found in non-*Enterobacteriaceae* species, such as *Shewanella*, *Acinetobacter*, and *Pseudomonas*. The coliform species harboring resistance genes would trigger a positive coliform water test, which would theoretically be followed by attempts to remediate the contamination issue; however non-*Enterobacteriaceae* species harboring ESBL and carbapenemase genes would evade detection by the most commonly used coliform screening methods resulting in “silent” dissemination of these genes via tap water that meets all current regulatory requirements.

Studies reporting ESBL- and carbapenemase-producing bacteria in drinking water in high-income countries are extremely rare. CTX-M-producing *E. coli* was discovered in drinking water in France in a single water sample^10^, and carbapenemase-producing *Serratia fonticola* has previously been reported in drinking water from Portugal^32^, as have non-fermenting intrinsic carbapenemase-producers^13^. To our knowledge, this is the first extensive study of drinking water from multiple regions in a high income country that has revealed the geographically widespread distribution of ESBL- and carbapenemase-producing isolates of serious clinical concern. Our findings suggest that community exposure to these organisms may be more common than currently realized, and consequently, their prevalence in the general population may be underestimated.

In this era of increasing antimicrobial resistance, it is critical to determine the public health significance of antibiotic resistance genes in community drinking water regardless of country income-level classification. Public water systems provide an effective means by which pathogens and antibiotic-resistant organisms can be transmitted to large segments of the population. In high-income countries, approaches such as increased consumption of bottled water are not adequate solutions, as bottled water is often derived from the same sources as tap water, and may also contain trace levels of total coliform bacteria^33^. More research is needed to better characterize the problem, understand the associated risk, and devise solutions to combat antimicrobial-resistant bacteria in tap water^2^.

## Supplementary information

Supplemental Table 1

## Data Availability

ESBL or carbapenemase gene sequence data that support the findings of this study have been deposited into GenBank with the accession numbers listed in Table 1. The data that support the descriptive statistical analyses of sample characteristics and resistance gene presence are available from the corresponding author upon reasonable request.
