# Supplementary material for: Multi-state study of Enterobacteriaceae harboring extended-spectrum beta-lactamase and carbapenemase genes in U.S. drinking water
Source: Sci Rep. 2019 Mar 8;9:3938. doi: 10.1038/s41598-019-40420-0 (PMC6408426; doi:10.1038/s41598-019-40420-0)
Supplement: Supplementary file 1 — Supplemental Table 1 [file 41598_2019_40420_MOESM1_ESM.docx]

Multi-state study of *Enterobacteriaceae* harboring extended-spectrum beta-lactamase and carbapenemase genes in U.S. drinking water

WD Tanner, JA VanDerslice, RK Goel, MK Leecaster, MA Fisher, J Olstadt, CM Gurley, AG Morris, KA Seely, L Chapman, M Korando, KA Shabazz, A Stadsholt, J VanDeVelde, E Braun-Howland, C Minihane, PJ Higgins, M Deras, O Jaber, D Jette, AV Gundlapalli

Supplemental Table 1. Primer sequences used in the ESBL and carbapenemase gene PCR assays

| PCR protocol | Primer | Primer sequence (5’ -> 3’) | Amplicon size | Reference |
| --- | --- | --- | --- | --- |
| ESBL Gene Multiplex | *bla*_TEM_-F  *bla*_TEM_-R | tcgccgcatacactattctcagaatga  acgctcaccggctccagatttat | 445 bp | Monstein, et al. ^16^ |
| ESBL Gene Multiplex | *bla*_CTX-M_-F  *bla*_CTX-M_-R | atgtgcagyaccagtaargtkatggc  tgggtraartargtsaccagaaycagcgg | 593 bp | Monstein, et al. ^16^ |
| ESBL Gene Multiplex | *bla*_SHV_-F  *bla*_SHV_-R | atgcgttatattcgcctgtg  tgctttgttattcgggccaa | 747 bp | Monstein, et al. ^16^ |
| Carbapenemase Gene Multiplex | *bla*_OXA-48_-F  *bla*_OXA-48_-R | GCGTGGTTAAGGATGAACAC  CATCAAGTTCAACCCAACCG | 438 bp | Poirel, et al. ^17^ |
| Carbapenemase Gene Multiplex | *bla*_NDM_-F  *bla*_NDM_-R | GGTTTGGCGATCTGGTTTTC  CGGAATGGCTCATCACGATC | 621 bp | Poirel, et al. ^17^ |
| Carbapenemase Gene Multiplex | *bla*_KPC_-F  *bla*_KPC_-R | CGTCTAGTTCTGCTGTCTTG  CTTGTCATCCTTGTTAGGCG | 798 bp | Poirel, et al. ^17^ |
| CTX-M Group-Specific Gene Multiplex | CTX-M-Group 1-F  CTX-M-Group 1-R | AAAAATCACTGCGCCAGTTC  AGCTTATTCATCGCCACGTT | 415 bp | Woodford, et al. ^19^ |
| CTX-M Group-Specific Gene Multiplex | CTX-M-Group 2-F  CTX-M-Group 2-R | CGACGCTACCCCTGCTATT  CCAGCGTCAGATTTTTCAGG | 552 bp | Woodford, et al. ^19^ |
| CTX-M Group-Specific Gene Multiplex | CTX-M-Group 9-F  CTX-M-Group 9-R | CAAAGAGAGTGCAACGGATG  ATTGGAAAGCGTTCATCACC | 205 bp | Woodford, et al. ^19^ |
| CTX-M Group-Specific Gene Multiplex | CTX-M-Group 8-F  CTX-M-Group 8-R | TCGCGTTAAGCGGATGATGC  AACCCACGATGTGGGTAGC | 666 bp | Woodford, et al. ^19^ |
| CTX-M Group-Specific Gene Multiplex | CTX-M-Group 25-F  CTX-M-Group 25-R | GCACGATGACATTCGGG  AACCCACGATGTGGGTAGC | 327 bp | Woodford, et al. ^19^ |
| *bla*_SHV_ gene | Full_SHV-F  Full_SHV-R | GCCCGGGTTATTCTTATTTGTCGC  TCTTTCCGATGCCGCCGCCAGTCA | 1017 bp | Nuesch-Inderbinen, et al. ^20^ |
| *bla*_CTX-M_ gene | Full_CTX-M-1-F  Full_CTX-M-1-R | GACTATTCATGTTGTTGTTATTTC  TTACAAACCGTTGGTGACG | 923 bp | Mena et al. ^21^ |
| *bla*_TEM_ gene | Full_TEM-F  Full_TEM-R | TTCTTGAAGACGAAAGGGC  ACGCTCAGTGGAACGAAAAC | 1150 bp | Brinas et al. ^22^ |
| *bla*_OXA-48_-type gene | Full_OXA-48-F  Full_OXA-48-R | TTGGTGGCATCGATTATCGG  GAGCACTTCTTTTGTGATGGC | 743 bp | Poirel, et al ^23^ |
